# Initiation Patterns and Transitions Among Adults Using Stimulant Drugs: Latent Transition Analysis

**DOI:** 10.2196/46747

**Published:** 2023-10-05

**Authors:** Joshua C Black, Hannah L Burkett, Karilynn M Rockhill, Richard Olson, Richard C Dart, Janetta Iwanicki

**Affiliations:** 1 Rocky Mountain Poison and Drug Safety Denver Health and Hospital Authority Denver, CO United States

**Keywords:** stimulant misuse, high dimensional analysis, latent transition analysis, general population, stimulant drug, drug misuse, overdose, behavioral trajectory, drug overdose, stimulant initiation, drugs, substance abuse, analysis

## Abstract

**Background:**

The fourth wave of the drug overdose epidemic in the United States includes increasing rates of stimulant-involved overdose. Recent studies of transitions leading to stimulant misuse have shown complex patterns that are not universally applicable because they have isolated individual populations or individual behaviors. A comprehensive analysis of transitions between behaviors and the associations with present-day problematic drug use has not been conducted.

**Objective:**

This study aims to determine whether adults from the general population who use stimulants initiate use through a heterogeneous combination of behaviors and quantify the association between these typologies with present-day problematic drug use.

**Methods:**

Individuals who have reported use of any stimulant in their lifetime were recruited from the 2021 Survey of Nonmedical Use of Prescription Drugs Program, a nationally representative web-based survey on drug use, to participate in a rapid follow-up survey about their past stimulant use. Individuals were asked which stimulants they used, the reasons for use, the routes of administration, and the sources of the stimulant. For each stimulant-related behavior, they were asked at what age, between 6 and 30 years, they initiated each behavior in a 6-year time window. A latent transition analysis was used to characterize heterogeneity in initiation typologies. Mutually exclusive pathways of initiation were identified manually by the researchers. The association of these pathways with present-day problematic drug use was calculated using logistic regression adjusted by the current age of the respondent.

**Results:**

From a total of 1329 participants, 740 (55.7%) reported lifetime prescription stimulant use and 1077 (81%) reported lifetime illicit stimulant use. Three typologies were identified. The first typology was characterized by illicit stimulant initiation to get high, usually via oral or snorting routes and acquisition from friends or family or a dealer (illicit experimentation). The second typology was characterized by low, but approximately equal probabilities of initiating 1-2 new behaviors in a time window, but no singular set of behaviors characterized the typology (conservative initiation). The third was characterized by a high probability of initiating many diverse combinations of behaviors (nondiscriminatory experimentation). The choice of drug initiated was not a strong differentiator. Categorization of pathways showed those who were only in an illicit experimentation status (reference) had the lowest odds of having severe present-day problematic drug use. Odds were higher for a conservative initiation-only status (odds ratio [OR] 1.84, 95% CI 1.14-2.94), which is higher still for those moving from illicit experimentation to conservative initiation (OR 3.50, 95% CI 2.13-5.74), and highest for a nondiscriminatory experimentation status (OR 5.45, 95% CI 3.39-8.77).

**Conclusions:**

Initiation of stimulant-related use behaviors occurred across many time windows, indicating that multiple intervention opportunities are presented. Screening should be continued throughout adulthood to address unhealthy drug use before developing into full substance use disorders.

## Introduction

The drug overdose epidemic in the United States is constantly evolving, with new drugs emerging and old drugs resurfacing [1]. Recently, a resurgence of stimulant-involved overdose (eg, methamphetamine, amphetamine, and cocaine) [2], both with and without opioids [3], indicates the latest overdose wave involves concomitant use with stimulants, in addition to opioid-specific overdoses [4]. In 2020, nearly 489,000 people aged 12 years and older initiated nonpharmaceutical stimulant use, and 734,000 initiated prescription stimulant misuse [5]. Meanwhile, the dispensing of prescription stimulants is rising [6]. Physiologically, transitions from occasional stimulant use to addiction have been characterized by structural changes to the brain [7], while moderate to high doses of stimulants lead to euphoria, cognitive impairment, and potentially, psychosis [8]. Continued use of stimulants can lead to detrimental changes to neurological structures involved in impulse control, attention, disinhibition in social settings, and habit creation, potentially predicting transitions into use disorder through behavioral change [9]. Understanding behavioral changes would present opportunities for treatment and other interventions, and understanding drug-related behavior prior to progression to substance use disorder is a critical area that needs further study [10].

Recent assessments into nonmedical stimulant initiation have shown complex patterns that are not universally applicable to all individuals who nonmedically use stimulants. Adolescents who initiate stimulant misuse earlier in life are more likely to have future substance-related problems than those who initiate it later in life [11]. Initiation among college students peaks in certain months of the year [12] and it is correlated to high academic demand [13]. Among individuals entering treatment for opioid use disorder, the number of years between the first use of opioids and stimulants (or vice versa) has steadily decreased from 1991 to 2020 [14], indicating a faster progression through drug use trajectories. In a survey of individuals recruited from Reddit and reporting nonoral prescription stimulant misuse, misuse of prescription stimulants and marijuana preceded the first use of many nonpharmaceutical drugs such as cocaine, methamphetamine, and heroin [15]. In a different study, prescription stimulant misuse was not seen as an initiating factor, as it likely occurred after other drug use [16]. These studies have isolated individual populations (eg, college students) or individual behaviors (eg, initiation of any misuse) when examining transitions. A comprehensive analysis of transitions between behaviors and the associations with present-day problematic drug use has not been done.

Our goal is to determine whether adults from the general population who use stimulants follow a single behavioral pattern of initiation or whether initiation occurs through a heterogeneous combination of behaviors. Using a latent transition methodology, we categorized initiation into typologies, which represent different progressions of stimulant initiation. We also quantify the association between these typologies with present-day problematic drug use. We hypothesized that more than 1 initiation typology would be detected.

## Methods

### Data Source

A retrospective cross-sectional custom stimulant survey was conducted among adults in the United States who have reported any lifetime stimulant use. This custom stimulant survey was deployed in conjunction with the Researched Abuse, Diversion, and Addiction-Related Surveillance System routine web-based drug survey of the general adult population, the Survey of Non-Medical Use of Prescription Drugs (NMURx) Program, which has been shown to be valid [17] and reliable [18] against 3 national benchmark surveys on drug use and health.

### Parent Survey Description

The parent NMURx Program survey is a national sample selected from a web-based panel, which is a group of individuals willing to take surveys for modest compensation [17]. A survey administration company recruits panelists and administers the survey. Panelists were recruited through advertising, peer recruitment, and sponsored recruitment events, and panelist recruitment was conducted independently from the parent NMURx Program survey. Within the panelist group, participants for the parent NMURx Program survey were selected to be representative of all regions and with even distribution between male participants and female participants. Selected participants had the NMURx Program survey appear in their list of available surveys on the web-based portal hosted by the survey administration company. The parent NMURx Program survey asks about drug use for prescription and nonpharmaceutical stimulants (among other drug classes). Participant demographics, treatment history, and the Modified Drug Abuse Screening Test (DAST-10) [19] are also collected. The DAST-10 is a continuous score from a self-administered instrument for problematic drug use, and a score of 3 or larger (herein described as “severe problematic drug use”) is a suitable indicator for risk of substance use disorder [20]. Data were collected from August 27 to October 10, 2021, with an overall completion rate of 70% (42,616 initiated surveys and 30,006 completed surveys). The study was conducted in accordance with the CHERRIES (Checklist for Reporting Results of Internet E-Surveys) checklist [21] (Multimedia Appendix 1).

### Custom Stimulant Initiation Survey Description

The parent NMURx Program survey was used as a case-finding tool. Participants who reported any lifetime stimulant use, prescription or nonpharmaceutical, received a follow-up survey within 2 weeks of their responses to the parent NMURx Program survey. Data collection for the follow-up survey occurred from September 13 to November 1, 2021. The follow-up survey appeared in the eligible participants’ list of available surveys, where they could optionally fill the questionnaire for additional compensation. Eligible panelists were not specifically targeted with recruitment emails or other communication, beyond nonspecific reminders from the survey administration company that surveys were available for them. A total of 8812 respondents (29.4% of the NMURx Program parent survey) reported lifetime use of prescription or nonpharmaceutical stimulants making them eligible to be recontacted. A total of 1919 were recruited into the follow-up stimulant survey and 1329 (69.3% completion rate) completed the survey. It was assumed that nonrecruitment into the follow-up survey was not associated with initiation typologies.

The follow-up stimulant survey asked additional questions about the initiation of stimulant-related behavior. Participants were first required to reconfirm whether they have used any stimulant in their lifetime to continue. Questions included which stimulant drugs have been used and at what age the first use occurred. In addition, for each stimulant reported, questions included a series of 17 behavioral questions about the route of administration (eg, snorting), the reason for use (eg, used to get high), and the source of the drug (eg, obtained from a friend or family) and the age they first engaged in such behaviors. The earliest reported initiation age for each behavior was used for analysis. A list of drugs and behavioral questions is provided (Multimedia Appendix 2).

The quality of retrospective data is at risk from recall bias and could be incomplete [22]; however, calendar instruments have been shown to mitigate this bias [23,24]. A calendar instrument was used that primed participants with life events. Participants were asked to provide 5 life events (eg, purchasing their first car) and the age those events occurred. When asked about drug-related ages of initiation, the responsive design elements of the survey used the life events and ages to aid in recall by being visible to the respondent, while drug behavior questions were asked. A screenshot of the calendar instrument used is included (Multimedia Appendix 2). A last question asking whether individuals’ answers should be trusted was used as exclusion criteria (if answered “No”) to reduce residual measurement bias; this has been shown to parsimoniously remove “careless” responses [25]. It was assumed that all responses were accurate without residual recall bias after exclusions were applied.

### Ethical Considerations

The NMURx Program study protocol was reviewed and approved by the Colorado Multiple Institutional Review Board prior to data being collected (#16-0922). The follow-up survey was approved by the Office of Management and Budget Paper Reduction Act review on July 13, 2021 (#0910-0847). Participants consented to be surveyed for both the parent survey and the custom stimulant survey. Data privacy is protected by a National Institutes of Health Certificate of Confidentiality.

### Latent Transition Analysis

A latent transition analysis (LTA) was conducted to identify distinct typologies and transitions between typologies [26]. Briefly, this exploratory approach assumes that participants transition between latent status, where a status represents a subgroup of participants with a similar set of initiation behaviors. The reported age of initiation age was used to construct a retrospective longitudinal trajectory of behaviors for each person. Six-year age windows were used, 6-11, 12-17, 18-23, 24-29, and 30+ years, to establish categorical transition windows. An initial latent class analysis was conducted to identify important indicators, where subsequent models collapsed or removed indicators to improve model performance and interpretability, as has been done previously [27]. Item-response probabilities were defined by the ρ parameters, which indicate the probability a person in a latent status would engage in a behavior. It was assumed that all initiation status were possible at all ages, and there were no time-varying changes in how statuses were defined.

A series of LTA models were fit to determine the optimal number of latent statuses. Models with 1 through 6 statuses were considered; the most parsimonious, based on the smallest Bayesian Information Criterion, and an interpretable model was selected. When 2 models were similarly interpreted, the most parsimonious model with fewer statuses was preferred. Finally, once the model was identified, mutually exclusive pathways of initiation were identified manually by the researchers. The association of these pathways with present-day severe DAST-10 scores (≥3) was calculated using logistic regression adjusted by the current age of the respondent. All analyses were conducted in SAS (version 9.4; SAS Institute).

## Results

### Descriptive Statistics

Within the follow-up sample of 1329, 740 (55.7%) participants confirmed lifetime use of a prescription stimulant and 1077 (81%) participants confirmed lifetime use of a nonpharmaceutical stimulant. Table 1 outlines participant characteristics by age of stimulant initiation. A total of 463 (34.8% of the entire sample) initiated before age 18 years of age, with 111 (8.4% of the entire sample) initiating from 6 to 11 years. Regardless of initiation ages, most initiated illicit stimulant use first or only used illicit stimulants, although the use of prescription drugs first was much higher in those who initiated after 22 years of age. Individuals initiating stimulant use before 23 years of age had slightly higher DAST-10 scores and were more likely to have used other drugs in their lifetime.

**Figure 1:**
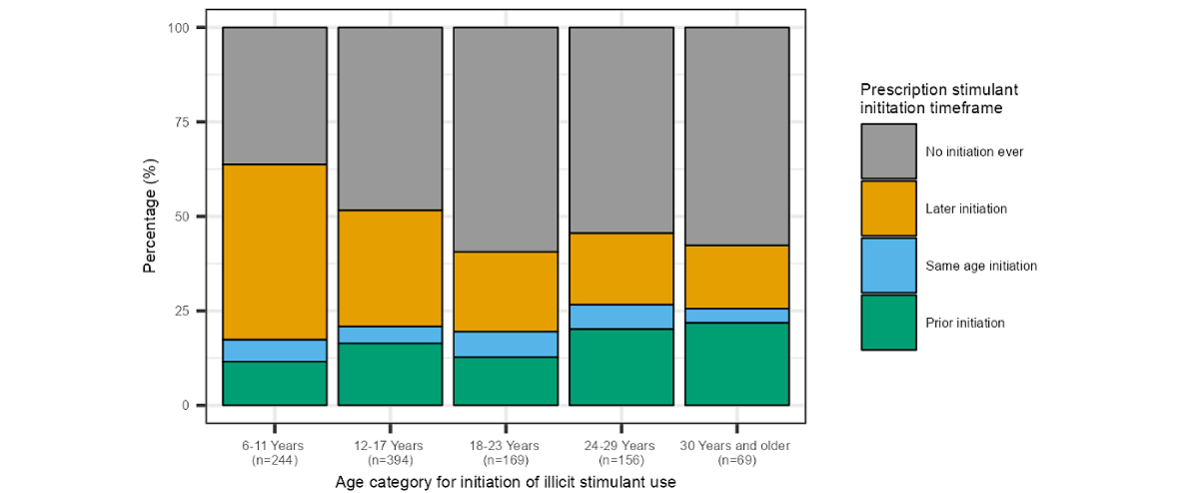
Prescription stimulant initiation relative to nonpharmaceutical initiation.

Figure 1 shows the timing of prescription stimulant initiation stratified by the age of initiation of nonpharmaceutical stimulant use (n=1077, 81%). Regardless of what age nonpharmaceutical stimulant use was initiated, less than a quarter of participants indicated prescription stimulant use came before nonpharmaceutical stimulant use. The later in life an individual initiated nonpharmaceutical stimulant use, the more likely they were to have ever used a prescription stimulant.

**Table 1:** Sample characteristics stratified by age of stimulant initiation.

Characteristic		Respondent age at initiation of stimulant use		
		<18 Years (n=463)	18-23 Years (n=421)	>23 Years (n=445)
**Sex, n (%)**				
	Male	279 (60.3)	250 (59.4)	247 (55.5)
	Female	184 (39.7)	171 (40.6)	198 (44.5)
**Age at survey completion (years)** **, n (%)**				
	18-24	20 (4.4)	12 (2.9)	Suppressed
	25-29	24 (5.3)	12 (2.9)	Suppressed
	30-39	142 (31.4)	90 (21.8)	93 (20.9)
	40-49	84 (18.6)	107 (25.9)	106 (23.9)
	50-59	84 (18.6)	85 (20.6)	86 (19.4)
	60+	98 (21.7)	107 (25.9)	154 (34.7)
**Census region, n (%)**				
	Northeast	82 (17.7)	73 (17.3)	67 (15.1)
	Midwest	97 (20.9)	88 (20.9)	77 (17.3)
	South	172 (37.1)	162 (38.5)	186 (41.8)
	West	112 (24.2)	98 (23.3)	115 (25.8)
**Race,^a^ n (%)**				
	American Indian or Alaska Native	19 (4.1)	20 (4.7)	15 (3.4)
	Asian	24 (5.2)	13 (3.1)	11 (2.5)
	Black or African American	32 (6.9)	34 (8.1)	30 (6.7)
	White	393 (84.9)	365 (86.7)	397 (89.2)
	Other	16 (3.5)	9 (2.1)	7 (1.6)
**Ethnicity, n (%)**				
	Hispanic or Latino	59 (12.7)	34 (8.1)	51 (11.5)
	Not Hispanic or Latino	404 (87.3)	387 (91.9)	394 (88.5)
**Type of stimulant initiation, n (%)**				
	Only used prescription or used first	25 (6.2)	12 (3.1)	89 (24.3)
	Only used illicit or used first	380 (93.6)	379 (96.7)	271 (74)
**DAST-10^b^ level, n (%)**				
	Low level or none reported (0-2)	342 (73.9)	309 (73.4)	354 (79.5)
	Moderate to severe level (3-10)	121 (26.1)	112 (26.6)	91 (20.4)
**Drug use history, n (%)**				
	Lifetime nonmedical use of any prescription pain reliever	183 (39.52)	143 (33.97)	122 (27.42)
	Lifetime nonmedical use of any prescription sedative	137 (29.6)	113 (26.8)	93 (20.9)
	Lifetime use of nonstimulant nonpharmaceutical drugs^c^	280 (60.48)	261 (62)	175 (39.3)

^a^Estimates among race categories may not sum to 100%.

^b^DAST-10: Drug Abuse Screening Test.

^c^Includes lifetime use of nonpharmaceutical forms of any of fentanyl, gamma-hydroxybutyrate, heroin, lysergic acid diethylamide, mescaline, phencyclidine, or psilocybin.

### Latent Transition Analysis

#### Model Overview

**Figure 2:**
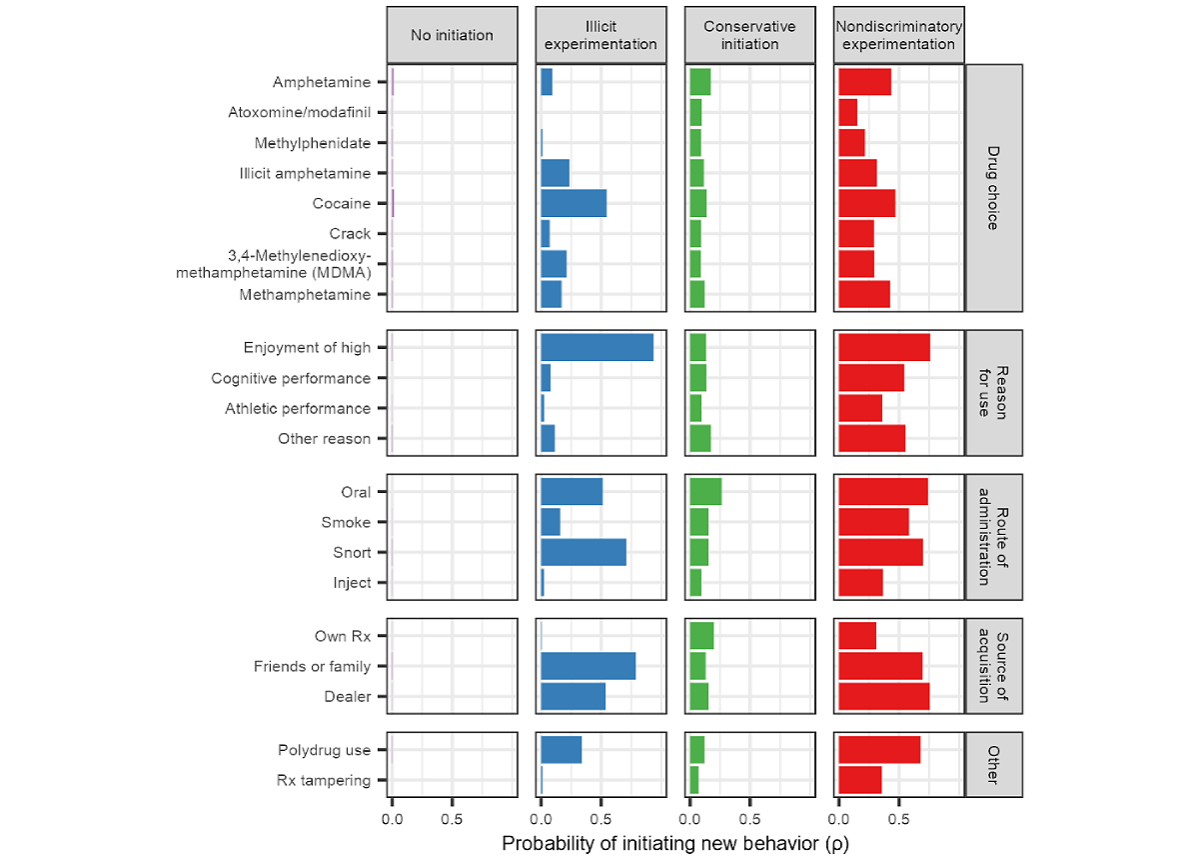
Item response probabilities of latent statuses.

The selected latent transition model based on patterns of initiation had 4 statuses which were held constant across the 5 age windows, as shown in Figure 2. Item-response probabilities (ρ parameters) are visually provided in Figure 2, while the values are provided in Multimedia Appendix 3. Based on an analysis of the behaviors associated with each status, the following names were selected: No initiation, illicit experimentation, conservative initiation, and nondiscriminatory experimentation. Brief descriptions are provided as follows.

#### No Initiation

This status was primarily defined by no initiation of new behaviors, and it was the predominant latent status over time. Participants in this status were not starting new behaviors (ρ near 0), although it is possible they could have continued behaviors they had initiated previously.

#### Illicit Experimentation

This status was characterized primarily by illicit stimulant initiation (usually cocaine, ρ=0.55) with a small probability of prescription amphetamine initiation (ρ=0.09). However, prescription amphetamine initiated in this status had a near zero probability of being acquired from their own prescription (ρ<0.01), indicating that any prescription amphetamine initiation that occurs in this status is nonmedical use. This status was also strongly characterized by initiating snorting stimulants (ρ=0.71) or oral use (ρ=0.51), new acquisition from friends or family members (ρ=0.79) or a dealer (ρ=0.54), and initiation of use to get high (ρ=0.94).

#### Conservative Initiation

This status was defined by low but approximately equal probabilities of initiating any use across all stimulant drugs (ρ values approximately 0.10). Furthermore, participants in this status were likely to initiate 1 or 2 new behaviors such as snorting (ρ=0.15) or obtaining the drug from a friend or family member (ρ=0.13). Notably, no singular set of reasons for use, routes of administration, or source of the drug strongly characterized this status. Rather, this status was characterized by participants selecting a small number of new behaviors to try during each age window.

#### Nondiscriminatory Experimentation

This status was characterized by a modest to high probability of engaging in multiple new behaviors across drugs, reasons, routes, and sources. Initiation of nonoral routes (ρ>0.50); initiation of use to get high (ρ=0.76), for cognitive performance (ρ=0.54), athletic performance (ρ=0.36); and polydrug use (ρ=0.68) were very likely in this status.

#### Latent Status Transitions

**Figure 3:**
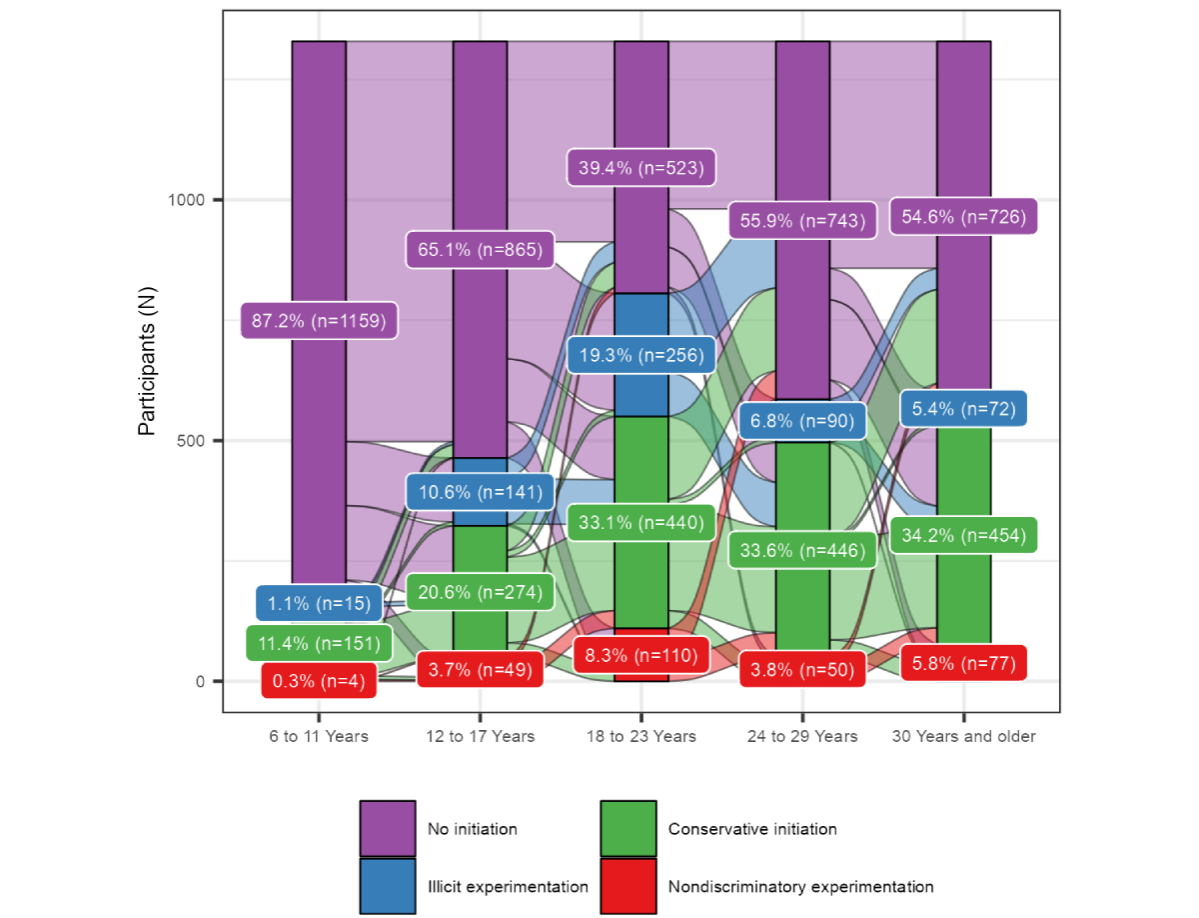
Transition probabilities and prevalence of latent statuses over time.

Figure 3 presents the movement of respondents between latent statuses across age windows as a Sankey plot. The width of the flow represents the percentage of individuals progressing between initiation statuses. No initiation latent status was the highest prevalence across all age windows, indicating that in most age windows, participants were not initiating new drug use behaviors. During the 6-11–year age window, the percentage of the No initiation status was the largest, with a small prevalence of conservative initiation, likely from initiation of medical use of stimulant drugs. Entering the 12-17–year age window, participants who were not initiating had a 14% probability of transitioning to the conservative initiation status and an 11% probability of transitioning into illicit experimentation. Entering the 18-23–year age window, the highest probability of progressing into the 2 Experimentation status was seen from the No initiation status. Entering the 24-29–year age window, those within the illicit experimentation had a 51% probability of transitioning into conservative initiation and a 48% probability of transitioning into No initiation. In the 24-29 and the 30 years and older age windows, transitions were primarily between conservative initiation and No initiation. During these 24 years and older age windows, a large percentage of respondents are characterized by no new initiation, indicating they were not trying new behaviors. All LTA item-response probabilities and transition probabilities are provided in Multimedia Appendix 3.

### Pathways of Stimulant Use Progression

Four common initiation pathways across the ages were identified to parsimoniously define how individuals progressed through different behaviors related to stimulant use. Respondents were classified as (1) only transitioning to the conservative initiation status (n=481, 36%), (2) only transitioning to the illicit experimentation status (n=264, 21%), (3) transitioning between illicit experimentation and conservative initiation (n=250, 19%), and (4) transitioning to the nondiscriminatory experimentation status, regardless of any other status (n=288, 22%). Notably, it was extremely rare (n=46, 3%) for participants to transition from conservative initiation to illicit experimentation (seen in Figure 3 as very little flow from green to blue).

The odds of having present-day severe DAST-10 scores significantly differed by the initiation pathway participants followed through time, even after adjusting for birth cohort (Table 2). Those with an initiation pathway involving nondiscriminatory initiation had 5.45 (95% CI 3.39-8.77) times the odds of a severe present-day DAST-10 score compared to those within the only illicit experimentation pathway. Those who progressed from illicit experimentation to conservative initiation had 3.50 (95% CI 2.13-5.74) times the odds of a severe DAST-10 score compared to those within the only illicit experimentation pathway. Those with only conservative initiation had 1.84 (95% CI 1.14-2.94) times the odds of a severe DAST-10 score as compared to those with only illicit experimentation.

The amount of time spent initiating new stimulant use differed between pathways. Among participants who only transitioned into the conservative initiation status, on average, they spent 2 age windows in this status. Among participants who only transitioned into the illicit experimentation status, on average, spent 1 age window in this initiation status. Therefore, those who only initiated illicit experimentation tended to do so in only 1 period of their life, while those who initiated more conservatively continued to initiate new behaviors across multiple periods in their lives.

**Table 2:** The odds of having present-day severe Modified Drug Abuse Screening Test (DAST-10) scores by initiation pathway.

Characteristic			Odds ratio (95% CI)
**Progression category (Ref=Only illicit experimentation)**			
	Only conservative initiation	1.84 (1.14-2.94)	
	Illicit experimentation leading to conservative initiation	3.50 (2.13-5.74)	
	Any nondiscriminatory initiation	5.45 (3.39-8.77)	
**Birth cohort (Ref=50+ Years) (years)**			
	18-29	1.86 (1.09-3.18)	
	30-49	2.03 (1.51-2.72)	

## Discussion

### Principal Findings

Stimulant initiation is not homogeneous. This study uniquely identified subtypes of initiation in use rather than static classes of how stimulants are used. There were three subtypes of initiation characterizing (1) a slower, more conservative initiation pattern engaging in few behaviors in the age window, (2) a distinct initiation of illicit drugs to get high with oral or snorting use, and (3) a more varied, less discriminating initiation pattern engaging in many behaviors in the age window. None of the initiation subtypes were predominantly defined by the drug used, although preferences such as the preference of illicit experimentation to initiate cocaine use were observed. The number and variety in behaviors initiated, including reason for use, route of administration, and sourcing the drug, was a stronger differentiating factor than the choice of drug. Findings are consistent with other literature showing those who initiate stimulant use earlier in life have more substance-related problems [11].

### Implications for Intervention

The progression of individuals through stimulant use behaviors is heterogeneous, with potential consequences for problematic drug use later in life. With the recognition that earlier stages of drug use need to be targeted for clinical intervention [10], screening for and discussing patients’ behavioral patterns could be a way for clinicians to address drug use before patients develop more severe substance use disorders. The results presented here show that individuals who protract their initiation of nonmedical use behaviors, including potentially nonmedical use of prescription stimulants, across many years are at higher risk of present-day problematic drug use than those who had a single initiation period involving snorting or oral use of nonpharmaceutical stimulants to get high. It is commonly known that initiation of stimulant nonmedical use often occurs during early adulthood [11], and these results confirm that. However, these results also show that initiation continued for many into the second half of their 20s and beyond. The conservative initiation status, often occurring during at least 2 time windows, was predictive of present-day problematic drug use, and therefore could benefit from intervention at multiple time points in a patient’s life if these behaviors are detected by health care professionals. Screening for unhealthy drug use is recommended for all adults [28], and tools such as the Screening, Brief Intervention, and Referral to Treatment approach [29] could offer prompts for health care professionals to begin discussions with their patients.

Another high-risk pattern identified that signals potential problematic use was nondiscriminatory experimentation. Study participants with this initiation pattern, consisting of one-fifth of all those studied, had 5.45 times greater odds of a severe DAST-10 score in the present day. Major features of polysubstance use and use by nonoral routes suggest high risk for other diagnoses including infectious disease exposures and use disorders beyond stimulants alone. These individuals may benefit from early intervention if this pattern is screened for and identified, both through harm reduction measures and other medical and psychiatric evaluation and treatment options.

Notably, prescription stimulant nonmedical use did not emerge as a separate latent status nor was it common for individuals to use prescription stimulants before nonpharmaceutical stimulants, indicating that behavior is not a differentiating factor in determining lifetime stimulant use progression. This could be an important distinction for the progression of stimulant nonmedical use from what has been observed historically for opioid nonmedical use. Although an LTA analysis was not conducted on opioids, past work has shown prescription opioid use often precedes nonpharmaceutical opioid use [30,31]. Despite the fact that those who use stimulants are approximately twice as likely to misuse them than those who use opioids [5], the results presented here show that initiation of prescription stimulant use first is uncommon, even as the likelihood of using a prescription stimulant has increased in younger birth cohorts.

### Strengths and Limitations

The primary strength of this study is that participants were sourced from a large general population survey, and not from specific subpopulations (eg, college students). This allowed a more comprehensive understanding of what subtypes of behaviors are present and how they changed over time. The study also proactively mitigated measurement bias using previously established methods to exclude careless respondents.

The study has 3 primary limitations. The first is recall bias. For some, participants were asked to recall behaviors from decades ago, which is likely incomplete or misremembered. However, the inclusion of a calendar tool mitigated recall bias. Second, the responding sample was skewed toward males, older individuals, White individuals, individuals from the western and southern regions, and higher-income individuals relative to the larger survey. However, the sample analyzed had similar overall problematic drug use scores to the larger survey, which could mitigate demographic-related bias. Third, while the study was recruited from a large diverse sample, the results were unweighted. Biases inherent in the self-selection, such as the relative poorer health of panelists [17], are uncorrected. Taken together, the low recruitment rate, demographic differences, and panel self-selection mean the results may not identify all possible initiation statuses. For example, unique pathways experienced by non-White participants or those from younger birth cohorts may be undetectable in this study. Statistical methods that correct for misrepresentation in follow-up surveys, such as the application of nested case-control designs [32], may enhance the detection of underrepresented pathways.

### Conclusions

Public health implications of this work include the importance of repeated screening for both nonmedical and nonpharmaceutical stimulant use and clarity on how different progressions might lead to future problematic behavior. In future work, models evaluating polysubstance initiation and longitudinal studies of trajectories will be crucial to understanding the role of stimulants within a broader model of substance use. While stimulant use is studied as a single phenomenon here, it is part of a much broader pattern of use of multiple substances.

## Appendix

Multimedia Appendix 1 CHERRIES Checklist.

Multimedia Appendix 2 Additional methodology description.

Multimedia Appendix 3 LTA parameters.

## Abbreviations

CHERRIES: Checklist for Reporting Results of Internet E-Surveys
DAST: Drug Abuse Screening Test
LTA: latent transition analysis
NMURx Program: Survey of Non-Medical Use of Prescription Drugs Program

## Data Availability

To protect participant privacy, row-level data cannot be made available.
