# Supplementary material for: Initiation Patterns and Transitions Among Adults Using Stimulant Drugs: Latent Transition Analysis
Source: J Med Internet Res. 2023 Oct 5;25:e46747. doi: 10.2196/46747 (PMC10587808; doi:10.2196/46747)

Multimedia Appendix 2: Additional Methods

Additional Methods:

There were 4 prescription stimulants and 6 nonpharmaceutical stimulants included on the follow-up stimulant survey. The following list shows the drugs included and examples provided to respondents (in italics). These items were randomized to each survey respondent.

- **Amphetamine made by a drug company** *such as Adderall®, Adderall XR®, Adzenys ER™, Adzenys XR-ODT™, Desoxyn®, Dexedrine®, Dyanavel® XR, Evekeo®, Evekeo® ODT™, Mydayis®, Vyvanse®, or other generics*
- **Atomoxetine** *such as Strattera® or other generics*
- **Methylphenidate** *such as Adhansia XR®, Aptensio XR®, Concerta®, Cotempla XR-ODT™, Daytrana®, Focalin®, Focalin XR®, Metadate CD®, Metadate® ER, Methylin™ ER, Quillichew ER™, Quillivant XR®, Ritalin®, Ritalin LA®, Ritalin SR®, or other generics*
- **Modafinil** *such as Nuvigil®, Provigil®, or other generics*
- **Amphetamine not made by a drug company** *(Bennies, Black Beauties, Crosses, Hearts, LA Turnaround, Speed, Truck Drivers, Uppers)*
- **Cathinones** *(mephedrone, bath salts)*
- **Cocaine Powder** *(Blow, Bump, Coke, Charlie, Flake, Snow, Toot)*
- **Crack Cocaine** *(Candy, Crack, Charlie, Rock)*
- **MDMA** *(Ecstasy, Molly, Adam, Clarity, Eve, Lover’s Speed, Peace, Uppers)*
- **Methamphetamine** *(Crank, Chalk, Crystal, Fire, Glass, Go Fast, Ice, Meth, Speed, Crystal Meth)*

There were 17 behaviors asked of the respondent to be placed on the timeline at age of first initiation if they reported ‘Yes’ to any of the following questions. These behaviors were asked for each stimulant and were randomly presented to respondents.

1. Have you ever swallowed the stimulant below?
2. Have you ever smoked the stimulant below?
3. Have you ever snorted the stimulant below?
4. Have you ever injected the stimulant below? Select yes or no for each.
5. Have you ever shared a needle with another person while injecting the stimulant below? (Note: only asked among those who reported injection)
6. Have you ever tampered with the prescription stimulant below before using it? Tampering could include crushing, chewing, heating, dissolving, or changing the drug in a way not directed by a healthcare professional.
7. Have you ever used the stimulant below to feel good or get high?
8. Have you ever used the stimulant below to improve professional or academic performance without being told to by a healthcare professional?
9. Have you ever used the stimulant below to improve athletic performance without being told to by a healthcare professional?
10. Have you ever used the stimulant below to lose weight?
11. Have you ever used the stimulant below at the same time as another drug? Please say no if you took a drug at the same time as another drug as directed by your healthcare provider.
12. Have you ever used the stimulant below as a replacement for another drug?
13. Have you ever received a prescription for yourself for the stimulant below?
14. Have you ever given away or sold the drugs from your prescription for the stimulant below? (Note: Only asked among those with their own prescription)
15. Have you ever received the stimulant below from a friend or family member?
16. Have you ever purchased the stimulant below from the Internet without a prescription?
17. Have you ever purchased the stimulant below from a dealer?

Respondents created a custom timeline based anchored at their current age which marked ages in which five major life events occurred. This method can help aid in recall but providing personalized context to events which occurred in their life during the age window of interest.


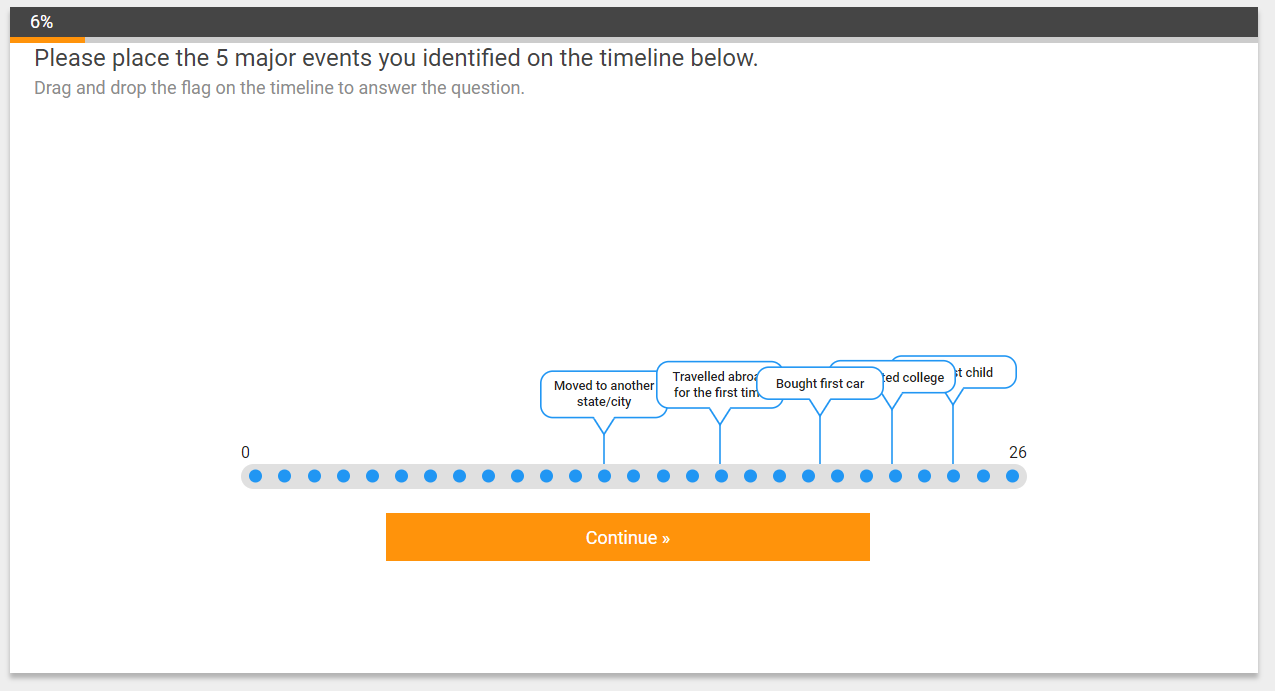


Stimulant use behaviors were then placed on the timeline based on age of initiation. The life events were visible to the respondent during this reporting.


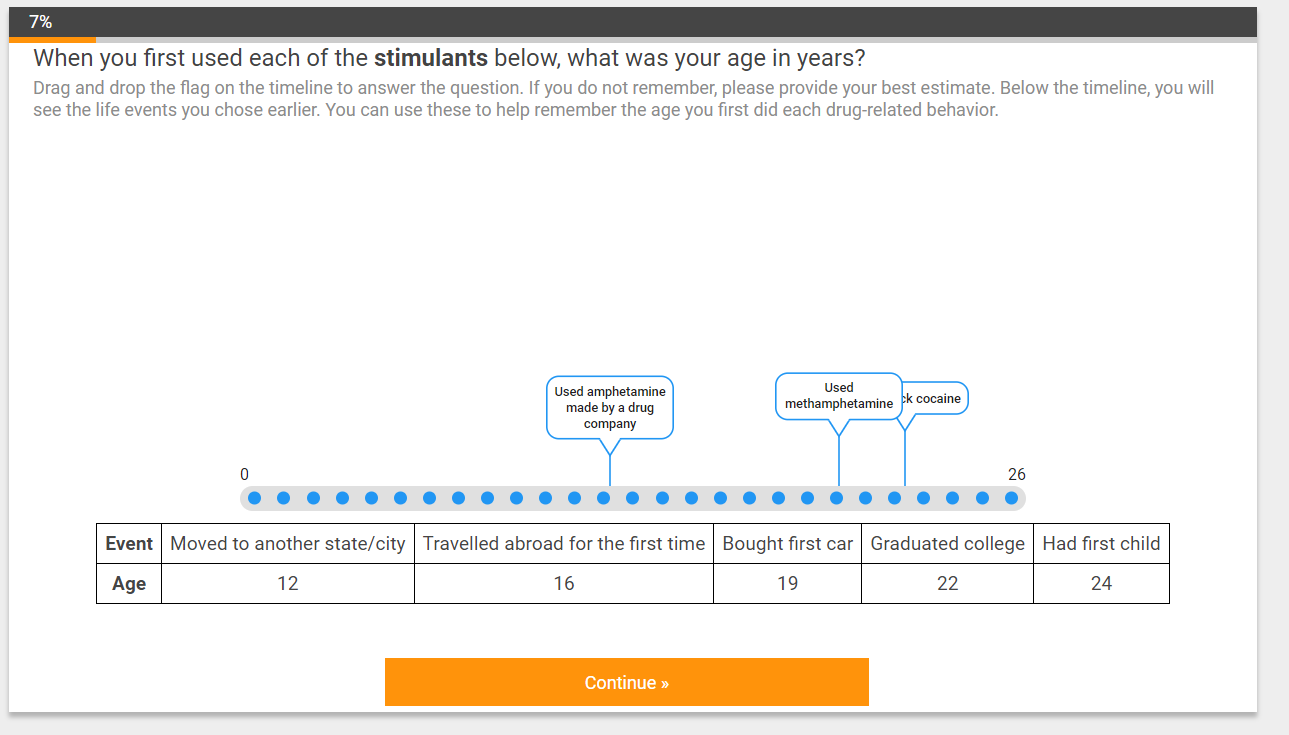

Supplement: Multimedia Appendix 2 [file jmir_v25i1e46747_app2.docx]
