# Supplementary material for: Initiation Patterns and Transitions Among Adults Using Stimulant Drugs: Latent Transition Analysis
Source: J Med Internet Res. 2023 Oct 5;25:e46747. doi: 10.2196/46747 (PMC10587808; doi:10.2196/46747)
Supplement: Multimedia Appendix 3 [file jmir_v25i1e46747_app3.docx]

Multimedia Appendix 3: LTA Parameters

**Supplemental Table 1: Item Response Probabilities (ρ Parameters) for Latent Statuses**

| **Indicator** | **No Initiation** | **Conservative Initiation** | **Illicit Experimentation** | **Non-Discriminatory Experimentation** |
| --- | --- | --- | --- | --- |
| **Drug Choice** | | | | |
| Amphetamine | <0.01 | 0.17 | 0.09 | 0.44 |
| Atomoxetine/Modafinil | <0.01 | 0.10 | <0.01 | 0.15 |
| Methylphenidate | <0.01 | 0.09 | 0.01 | 0.22 |
| Illicit Amphetamine | <0.01 | 0.12 | 0.23 | 0.32 |
| Cocaine | 0.014 | 0.14 | **0.55** | 0.47 |
| Crack | <0.01 | 0.09 | 0.07 | 0.29 |
| MDMA | <0.01 | 0.09 | 0.21 | 0.29 |
| Methamphetamine | <0.01 | 0.12 | 0.17 | 0.43 |
| **Reason for Use** | | | | |
| Enjoyment of High | <0.01 | 0.13 | **0.94** | **0.76** |
| Cognitive Performance | <0.01 | 0.14 | 0.08 | **0.54** |
| Athletic Performance | <0.01 | 0.10 | 0.03 | 0.36 |
| Other Reason | <0.01 | 0.17 | 0.12 | **0.55** |
| **Route of Administration** | | | | |
| Oral | <0.01 | 0.26 | **0.51** | **0.74** |
| Smoke | <0.01 | 0.15 | 0.15 | **0.58** |
| Snort | <0.01 | 0.15 | **0.71** | **0.70** |
| Inject | <0.01 | 0.09 | 0.02 | 0.36 |
| **Source of Acquisition** | | | | |
| Own Prescription^a^ | <0.01 | 0.20 | <0.01 | 0.31 |
| Friends or Family | <0.01 | 0.13 | **0.79** | **0.70** |
| Dealer | <0.01 | 0.15 | **0.54** | **0.75** |
| **Other Risk Behavior** | | | | |
| Polydrug Use | <0.01 | 0.12 | 0.34 | **0.68** |
| Prescription Tampering^a^ | <0.01 | 0.07 | 0.01 | 0.36 |

*Item response probabilities (ρ parameters) above 0.5 are bolded*

*^a^Question only asked among prescription stimulants*

**Supplemental Table 2: Transition Probabilities (τ Parameters) Between Latent Status Across the Age Windows**

|  | | **Ending Latent Status (τ)** | | | |
| --- | --- | --- | --- | --- | --- |
| **Age Windows of Transition** | **Starting Latent Status** | **No Initiation** | **Conservative Initiation** | **Illicit Experimentation** | **Non-Discriminatory Experimentation** |
| 6-11  into  12-17 | No Initiation | 0.71 | 0.14 | 0.11 | 0.04 |
|  | Conservative Initiation | 0.17 | 0.71 | 0.07 | 0.06 |
|  | Illicit Experimentation | 0.39 | 0.61 | <0.01 | <0.01 |
|  | Non-Discriminatory Experimentation | 0.36 | 0.28 | <0.01 | 0.36 |
| 12-17  into  18-23 | No Initiation | 0.48 | 0.16 | 0.28 | 0.09 |
|  | Conservative Initiation | 0.18 | 0.63 | 0.07 | 0.12 |
|  | Illicit Experimentation | 0.28 | 0.69 | <0.01 | 0.02 |
|  | Non-Discriminatory Experimentation | 0.27 | 0.73 | <0.01 | <0.01 |
| 18-23  into  24-29 | No Initiation | 0.66 | 0.16 | 0.15 | 0.03 |
|  | Conservative Initiation | 0.40 | 0.49 | 0.03 | 0.08 |
|  | Illicit Experimentation | 0.64 | 0.36 | <0.01 | <0.01 |
|  | Non-Discriminatory Experimentation | 0.49 | 0.50 | <0.01 | <0.01 |
| 24-29  into  30+ | No Initiation | 0.62 | 0.24 | 0.09 | 0.06 |
|  | Conservative Initiation | 0.44 | 0.46 | 0.02 | 0.08 |
|  | Illicit Experimentation | 0.48 | 0.51 | <0.01 | 0.02 |
|  | Non-Discriminatory Experimentation | 0.29 | 0.69 | <0.01 | 0.02 |
